# Immune-related adverse events and clinical outcomes in patients with solid tumors treated with adebrelimab: a real-world exploratory study

**DOI:** 10.3389/fonc.2026.1957193

**Published:** 2026-09-16

**Authors:** Yifan Chao, Fangyue Chang, Qianqian Li, Xiangyu Cheng, Yusheng Wang

**Affiliations:** 1 Department of Gastrointestinal Oncology, The First Hospital of Shanxi Medical University, Taiyuan, Shanxi, China; 2 Department of Gastroenterology, Shanxi Province Cancer Hospital, Taiyuan, Shanxi, China

**Keywords:** adebrelimab, immune-related adverse events, objective response rate, PD-L1 inhibitor, solid tumors

## Abstract

**Objective:**

To investigate the association between treatment efficacy and immune-related adverse events (irAEs) in patients with solid malignancies receiving adebrelimab.

**Methods:**

Clinical data from 46 patients with solid tumors who received adebrelimab between June 2023 and March 2026 were retrospectively collected. Patients were categorized as having experienced irAEs during treatment, and efficacy outcomes were compared between the irAE and non-irAE groups. Logistic regression analysis was used to evaluate the association between irAEs and objective response rate (ORR), whereas the Kaplan–Meier method, Cox regression, and time-dependent Cox models were used to assess the association between irAEs and progression-free survival (PFS).

**Results:**

Of the 46 patients, 40 (87.0%) experienced treatment-related adverse events (TRAEs), including 5 (10.9%) with grade 3–4 events. Eighteen patients (39.1%) experienced irAEs, most of which were grade 1–2, with a median onset time of 62 days (IQR, 42–163; range, 22–417 days). The overall ORR and disease control rate (DCR) were 37.0% and 87.0%, respectively. The ORR was higher in the irAE group than in the non-irAE group (61.1% vs. 21.4%, *P* = 0.012), whereas no significant difference in DCR was observed between the two groups (94.4% vs. 82.1%, *P* = 0.380). In univariate analysis, irAE occurrence was associated with ORR (OR = 5.762, 95% CI: 1.557–21.328; *P* = 0.009); however, after adjustment for the number of treatment cycles, this association did not reach statistical significance (OR = 3.847, 95% CI: 0.961–15.410; *P* = 0.057). Neither conventional Cox regression nor time-dependent Cox regression demonstrated a statistically significant association between irAE occurrence and PFS (HR = 1.906, 95% CI: 0.710–5.113, *P* = 0.200; HR = 2.444, 95% CI: 0.911–6.557, *P* = 0.076, respectively).

**Conclusion:**

Adebrelimab demonstrated an overall acceptable and manageable safety profile, with most irAEs being low-grade. Although irAE occurrence was associated with a higher ORR, this association was attenuated after adjustment for the number of treatment cycles, and no significant association between irAEs and PFS was observed. The current findings are insufficient to support irAEs as an independent predictor of adebrelimab efficacy, and further validation in larger, multicenter, prospective studies is warranted.

## 1 Introduction

Immune checkpoint inhibitors (ICIs) have dramatically changed how solid malignancies are treated in recent years (1, 2).ICIs improve antitumor immune responses and restore T-cell function by blocking the programmed cell death-1 (PD-1)/programmed cell death ligand 1 (PD-L1) signaling pathway (3). They have shown long-lasting antitumor activity in lung cancer, esophageal cancer, gastric cancer, and other solid tumors (4–7). But prolonged immune system activity can also cause harm to the body’s own tissues, which can result in irAEs (8). The epidermis, endocrine system, lungs, liver, and kidneys are just a few of the organ systems that irAEs can impact (9). Some severe irAEs may result in treatment cessation or even be life-threatening, despite the fact that the majority of irAEs are mild to moderate in severity and may be handled with established treatment protocols (8, 9). Therefore, enhancing the management of immunotherapy requires a detailed understanding of the features and clinical importance of irAEs.

Adebrelimab is a humanized PD-L1 monoclonal antibody that blocks the PD-1/PD-L1 signaling pathway to produce its anticancer effects (10). Adebrelimab’s therapeutic potential for additional solid tumors has been investigated in recent years, in addition to extensive-stage small cell lung carcinoma (11–13). However, there is still a lack of evidence on the characteristics and clinical significance of irAEs in adebrelimab-treated patients.

Previous studies have suggested that irAEs occurring during ICI therapy may be associated with improved tumor response and survival outcomes; however, whether irAEs can serve as reliable predictive markers of treatment efficacy remains controversial. Current evidence is derived mainly from studies of other PD-1/PD-L1 inhibitors, whereas data on adebrelimab remain limited, particularly in real-world patient populations (14–18).

Therefore, this retrospective study analyzed real-world clinical data to evaluate the safety profile and characteristics of irAEs in patients with solid tumors treated with adebrelimab. We further explored the associations between irAEs and ORR, DCR, and PFS, while also considering the potential influence of treatment exposure and the timing of irAE onset on these associations.

## 2 Materials and methods

### 2.1 Study design, population, and data source

Patients with solid tumors who were treated with adebrelimab at the First Hospital of Shanxi Medical University between June 2023 and March 2026 were sequentially recruited in this investigation, which used a retrospective cohort study design. The electronic medical record system, inpatient medical records, and outpatient follow-up records were the sources of clinical data. Data on baseline characteristics, tumor-related information, treatment plans, efficacy evaluations, and safety were gathered. Criteria for Inclusion: (1) Individuals whose solid tumors are malignant, as determined by cytology or histology; (2) Individuals who have undergone at least one course of treatment with adebrelimab; (3) Individuals who possess comprehensive baseline clinical data, such as baseline characteristics, treatment plans, response evaluations, and records of adverse events; and (4) Individuals who have finished at least one imaging-based response evaluation. Exclusion Standards: (1) Lack of important clinical information that makes it impossible to finish safety or efficacy evaluations; (2) A history of severe active infection, autoimmune illness, or long-term immunosuppressive medication before starting adebrelimab treatment; and (3) Absence of treatment-related adverse event records. In the end, 46 patients receiving adebrelimab treatment were included.

Patients were divided into the irAE group and non-irAE group based on whether they experienced at least one irAE during therapy. Treatment efficacy and survival outcomes were compared between the two groups. This study was approved by the Ethics Committee of the First Hospital of Shanxi Medical University (Ethics approval No. KYLL-2025-005).

### 2.2 Treatment regimens and evaluation of efficacy

Adebrelimab medication was administered to all patients, with particular dosage schedules established according to clinical practice recommendations, tumor type, disease stage, and past treatment history. Combination therapy with chemotherapy, radiation, or targeted therapy was administered to some patients based on their clinical treatment needs and disease features.

According to the Response Evaluation Criteria in Solid Tumors (RECIST) version 1.1, the response was divided into the complete response (CR), partial response (PR), stable disease (SD), and progressive disease (PD). The ORR was (CR+PR)/(CR+ PR+SD+PD) ×100%. The DCR was (CR+PR+ SD)/(CR+PR+SD+PD) ×100%. The PFS was defined as the time from the start of treatment to progression or death from any cause. The values of the patients who were lost to follow-up, and those who did not progress were treated as censored values. The censored time was the last follow-up that confirmed that the patients had neither progressed nor died.

### 2.3 Assessment of TRAEs and irAEs

The National Cancer Institute’s Common Terminology Criteria for Adverse Events (CTCAE) Version 5.0 was used to evaluate and grade the severity of both TRAEs and irAEs. TRAEs were defined as adverse events occurring during treatment that were considered, based on clinical records, to be potentially related to the overall anticancer therapy. Because most patients received adebrelimab in combination with chemotherapy and/or other anticancer therapies, some adverse events could not be clearly attributed to adebrelimab alone. Therefore, nonspecific hematologic and gastrointestinal toxicities were not considered adebrelimab-specific toxicities. In compliance with guidelines for the management of immune checkpoint inhibitor-related toxicities, irAEs were thoroughly evaluated based on patients’ clinical presentations, laboratory test results, and imaging findings. Endocrine toxicity (such as thyroid dysfunction and hypophysitis), immune-related pneumonia, hepatitis, nephritis, and skin toxicity were among the irAEs reported in this trial. When a patient had more than one irAE incident, the severity of the irAE was assessed using the patient’s highest CTCAE grade.

### 2.4 Follow-up

Patients were monitored through imaging examinations, inpatient medical records, and outpatient follow-up visits. Follow-up data included radiological assessments, survival outcomes, and disease status. Tumor response was evaluated based on imaging examinations, which were generally performed every 2–3 treatment cycles until disease progression, death, treatment discontinuation, or the last follow-up date. The data cutoff date for this study was June 6, 2026.

### 2.5 Statistical analysis

IBM SPSS Statistics 27.0 and R software were utilized for statistical analysis, and GraphPad Prism 10.6 was used for graphing. Following a normality test, continuous variables with a normal distribution were expressed as mean ± standard deviation ( $\bar{\text{x}}$ ± s), and an independent samples t-test was used to compare groups; those without a normal distribution were expressed as median (interquartile range, IQR) and examined using the Mann–Whitney U test. Frequency (%) was used to describe categorical variables, and Fisher’s exact test or the χ² test were used to compare groups. Odds ratios (ORs) and 95% confidence intervals (CIs) were calculated using univariate logistic regression analysis to assess the associations between individual variables and ORR. Variables with *P* < 0.10 in the univariate analyses were considered for inclusion in the multivariate logistic regression model using the Enter method. Given the limited number of response events in this study, the multivariate model included only irAE occurrence and the number of treatment cycles, and the results were interpreted as exploratory.PFS was analyzed using the Kaplan–Meier method and Cox proportional hazards regression, with results presented as hazard ratios (HRs) and 95% CIs. No conventional multivariate Cox regression was performed because none of the variables had a *P* < 0.10 in the univariate Cox analysis. Given the time-dependent nature of irAE occurrence, a time-dependent Cox proportional hazards model was further used to evaluate the association between irAE occurrence and PFS and to reduce potential immortal time bias arising from fixed-group classification. Patients were coded as irAE = 0 before the first occurrence of an irAE and as irAE = 1 from the time of the first irAE onward. Patients who did not experience an irAE were coded as irAE = 0 throughout follow-up. Because only 16 PFS events occurred in this study, the time-dependent Cox model included irAE as the sole time-dependent covariate to reduce model complexity and the risk of overfitting. The proportional hazards assumption was assessed using Schoenfeld residuals. All tests were two-sided, and *P* < 0.05 was considered statistically significant. Given the small sample size and limited number of outcome events, all regression analyses were interpreted as exploratory.

## 3 Results

### 3.1 Clinical characteristics of patients

This study included 46 patients with solid tumors who received adebrelimab treatment. Among them, 18 patients (39.1%) developed at least one irAE during treatment, whereas 28 patients (60.9%) did not experience any irAEs. No significant differences were observed between the irAE group and non-irAE group in terms of age, sex, primary tumor type, histological origin, disease stage, Ki-67 level, line of therapy, prior surgery, concomitant chemotherapy, targeted therapy, or radiotherapy (P > 0.05).A significant difference was observed in the number of treatment cycles between the two groups. Patients in the irAE group received more cycles of adebrelimab treatment than those in the non-irAE group [6 (5–8) cycles vs. 5 (2–6) cycles, P = 0.016] (**Table 1**).

**Table 1:** Baseline characteristics of participants.

Characteristics	irAE group(n=18)	Non-irAE group (n=28)	p value
Age, n (%)			
<65years	9(50.0)	10(35.7)	0.337
≥65years	9(50.0)	18(64.3)	
Sex, n (%)			
Male	15(83.3)	19(67.9)	0.315
Female	3(16.7)	9(32.1)	
Primary tumor type, n (%)			
Lung cancer	7(38.9)	6(21.4)	0.391
Esophageal cancer	7(38.9)	12(42.9)	
Other tumors	4(22.2)	10(35.7)	
Histological classification, n (%)			
Non-neuroendocrine origin	7(38.9)	13(46.4)	0.615
Neuroendocrine origin	11(61.1)	15(53.6)	
Clinical stage, n (%)			
I–III	11(61.1)	17(60.7)	0.979
IV	7(38.9)	11(39.3)	
Ki-67 level (%)	70(60–82.5)	70(60–80)	0.663
Treatment line, n (%)			
First-line	15(83.3)	23(82.1)	1.000
Second-line	3(16.7)	5(17.9)	
Treatment cycles	6(5–8)	5(2–6)	0.016
Previous surgery, n (%)	3(16.7)	5(17.9)	1.000
Combined with chemotherapy, n (%)	17(94.4)	26(92.9)	1.000
Combined with targeted therapy, n (%)	4(22.2)	5(17.9)	0.721
Combined with radiotherapy, n (%)	5(27.8)	7(25.0)	1.000

irAE, immune-related adverse events.

“Other tumors” included gastric cancer, pancreatic cancer, colorectal cancer, head and neck malignancies, and other rare tumors.

### 3.2 Analysis of treatment-related adverse events and immune-related adverse events

Among the 46 patients, 40 (87.0%) experienced at least one TRAE, and 5 (10.9%) experienced grade 3–4 TRAEs. The most frequently reported TRAEs were white blood cell count decreased (29/46, 63.0%), nausea (29/46, 63.0%), neutrophil count decreased (28/46, 60.9%), anemia (22/46, 47.8%), platelet count decreased (21/46, 45.7%), poor appetite (21/46, 45.7%), and fatigue (21/46, 45.7%) (**Table 2**). These results reflect toxicities observed throughout the overall treatment course and should not be interpreted as the incidence of adebrelimab-specific toxicities, as most patients received combination therapy.

**Table 2:** Treatment-related adverse events observed during adebrelimab-based therapy.

TRAE	Any grade, n (%)	Grade 3–4, n (%)
Total	40(87.0)	5(10.9)
White blood cell count decreased	29(63.0)	1(2.2)
Neutrophil count decreased	28(60.9)	1(2.2)
Platelet count decreased	21(45.7)	0
Anemia	22(47.8)	0
ALT increased	11(23.9)	0
AST increased	10(21.7)	0
GGT increased	5(10.9)	0
Bilirubin increased	9(19.6)	0
Poor appetite	21(45.7)	0
Alopecia	16(34.8)	0
Nausea	29(63.0)	0
Fatigue	21(45.7)	0
Vomiting	7(15.2)	0
Constipation	14(30.4)	0

TRAE, treatment-related adverse event; irAE, immune-related adverse events; ALT, alanine transaminase; AST, aspartate transaminase; GGT, gamma-glutamyl transferase.

During treatment, 18 patients (39.1%) developed at least one irAE, including 3 patients (6.5%) with grade 3–4 irAEs. Hypothyroidism was the most common irAE (8/46, 17.4%), followed by pruritus (4/46, 8.7%), rash (2/46, 4.3%), and hypophysitis (2/46, 4.3%). Additionally, hyperthyroidism, immune-related pneumonitis, immune-related hepatitis, and nephritis each occurred in one patient (2.2%) (**Table 3**; **Figure 1**). The median time to first onset of an irAE among the 18 patients who experienced irAEs was 62 days (IQR, 42–163 days; range, 22–417 days).All irAEs were managed with appropriate clinical interventions, and no deaths attributable to irAEs were observed. When analyzed by affected organ system, the median time to first onset of irAEs involving the endocrine, dermatologic, renal, pulmonary, and hepatic systems was 82.0, 48.0, 49.0, 191.0, and 191.0 days, respectively. The numbers of patients in the respective organ-system categories were not mutually exclusive because some patients experienced irAEs involving multiple organ systems (**Table 4**).

**Table 3:** Immune-related adverse events.

irAE	Any grade, n (%)	Grade 3–4, n (%)
Total	18(39.1)	3(6.5)
Hypothyroidism	8(17.4)	1(2.2)
Hyperthyroidism	1(2.2)	0
Pneumonitis	1(2.2)	1(2.2)
Rash	2(4.3)	0
Pruritus	4(8.7)	0
Hypophysitis	2(4.3)	1(2.2)
Hepatitis	1(2.2)	0
Nephritis	1(2.2)	0

irAE, immune-related adverse events.

**Figure 1:**
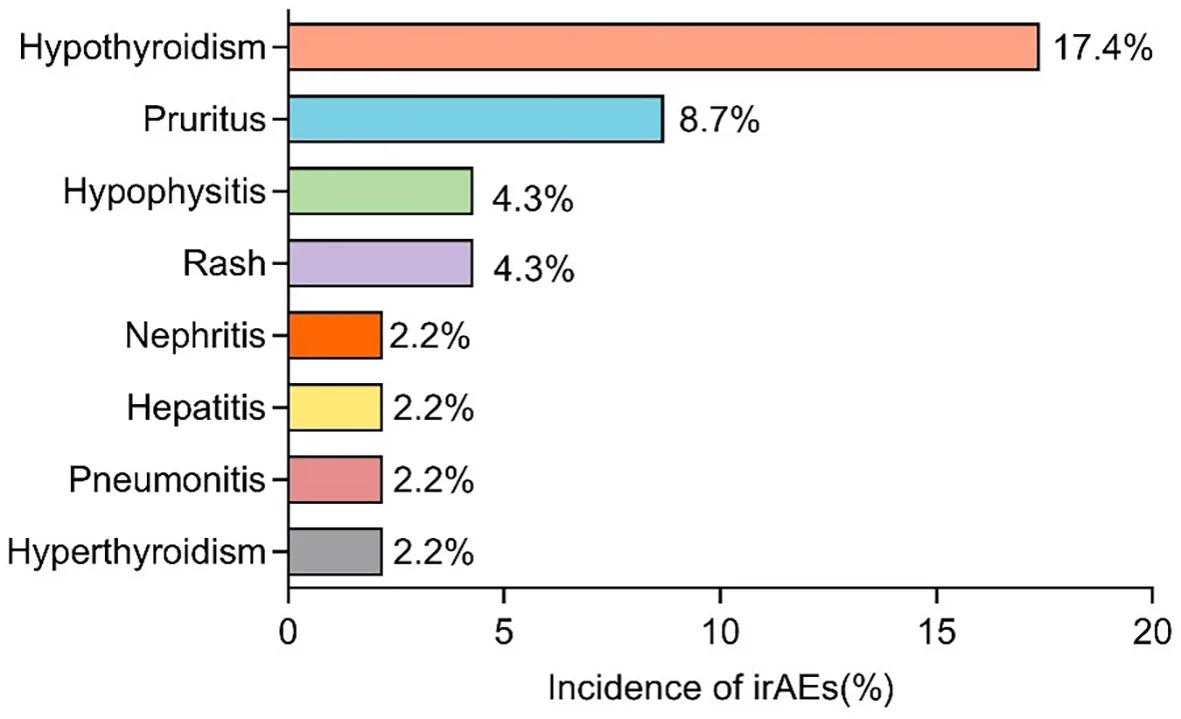
Types and incidence of immune-related adverse events (irAEs).

**Table 4:** Timing of irAE onset according to affected organ system.

Organ system	irAE type	n	Median time to onset(IQR), days	Range, days
Endocrine	Hypothyroidism, hyperthyroidism, hypophysitis	11	82.0(52.0–140.0)	22–417
Dermatologic	Pruritus, rash	6	48.0(35.25–58.0)	24–72
Renal	Nephritis	1	49.0	49–49
Pulmonary	Pneumonitis	1	191.0	191–191
Hepatic	Hepatitis	1	191.0	191–191

irAE, immune-related adverse events.

Some patients developed irAEs involving more than one organ system; therefore, patient counts across organ systems were not mutually exclusive. Onset time for each organ system was calculated according to the actual onset time of the corresponding irAE. IQR was not calculated for organ systems involving only one patient.

### 3.3 Association between irAEs and treatment response

Among the 46 patients, no patient achieved a CR. 17 patients (37.0%) achieved a PR, 23 (50.0%) had SD, and 6 (13.0%) experienced PD (**Figure 2**). The overall ORR was 37.0% (17/46), and the DCR was 87.0% (40/46). Patients were stratified into irAE group and non-irAE group based on irAE status. The ORR was significantly higher in the irAE group than in the non-irAE group. Eleven patients in the irAE group achieved PR, corresponding to an ORR of 61.1%, whereas 6 patients in the non-irAE group achieved PR, corresponding to an ORR of 21.4%. The difference in ORR between the two groups was statistically significant (P = 0.012). The DCRs were 94.4% and 82.1% in the irAE group and non-irAE group, respectively, with no significant difference observed (P = 0.380) (**Table 5**; **Figure 3**).

**Figure 2:**
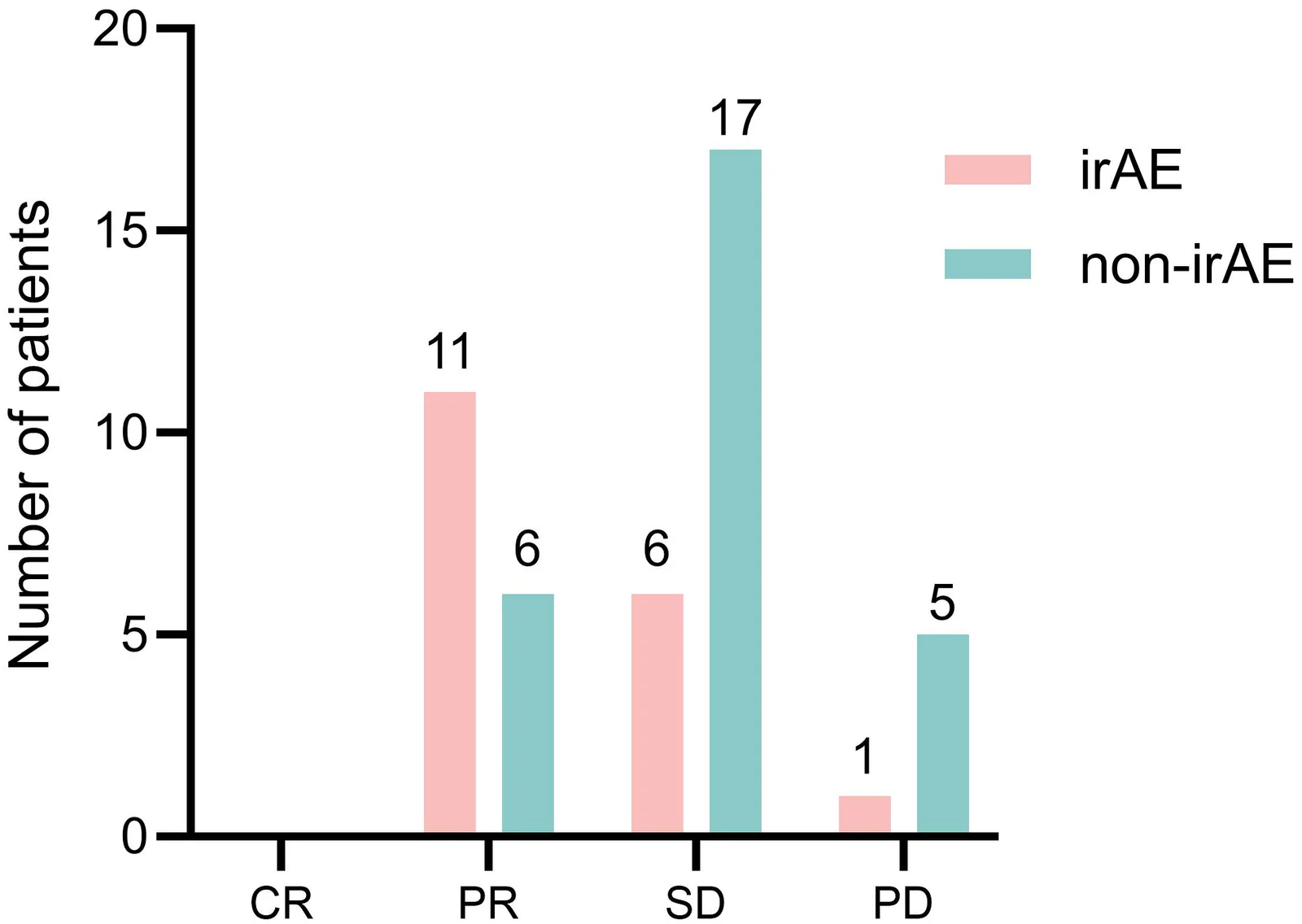
Distribution of best overall response categories according to irAE status.

**Table 5:** Impact of irAE on tumor response.

Effectiveness of treatment	All (n=46)	irAE group (n=18)	non-irAE group (n=28)	p value
CR	0	0	0	0.023
PR	17(37.0%)	11(61.1%)	6(21.4%)	
SD	23(50.0%)	6(33.3%)	17(60.7%)	
PD	6(13.0%)	1(5.6%)	5(17.9%)	
ORR%	17(37.0%)	11(61.1%)	6(21.4%)	0.012
DCR%	40(87.0%)	17(94.4%)	23(82.1%)	0.380

irAE, immune-related adverse events; CR, complete response; PR, partial response; SD, stable disease; PD, progressive disease; ORR, objective response rate; DCR, disease control rate.

**Figure 3:**
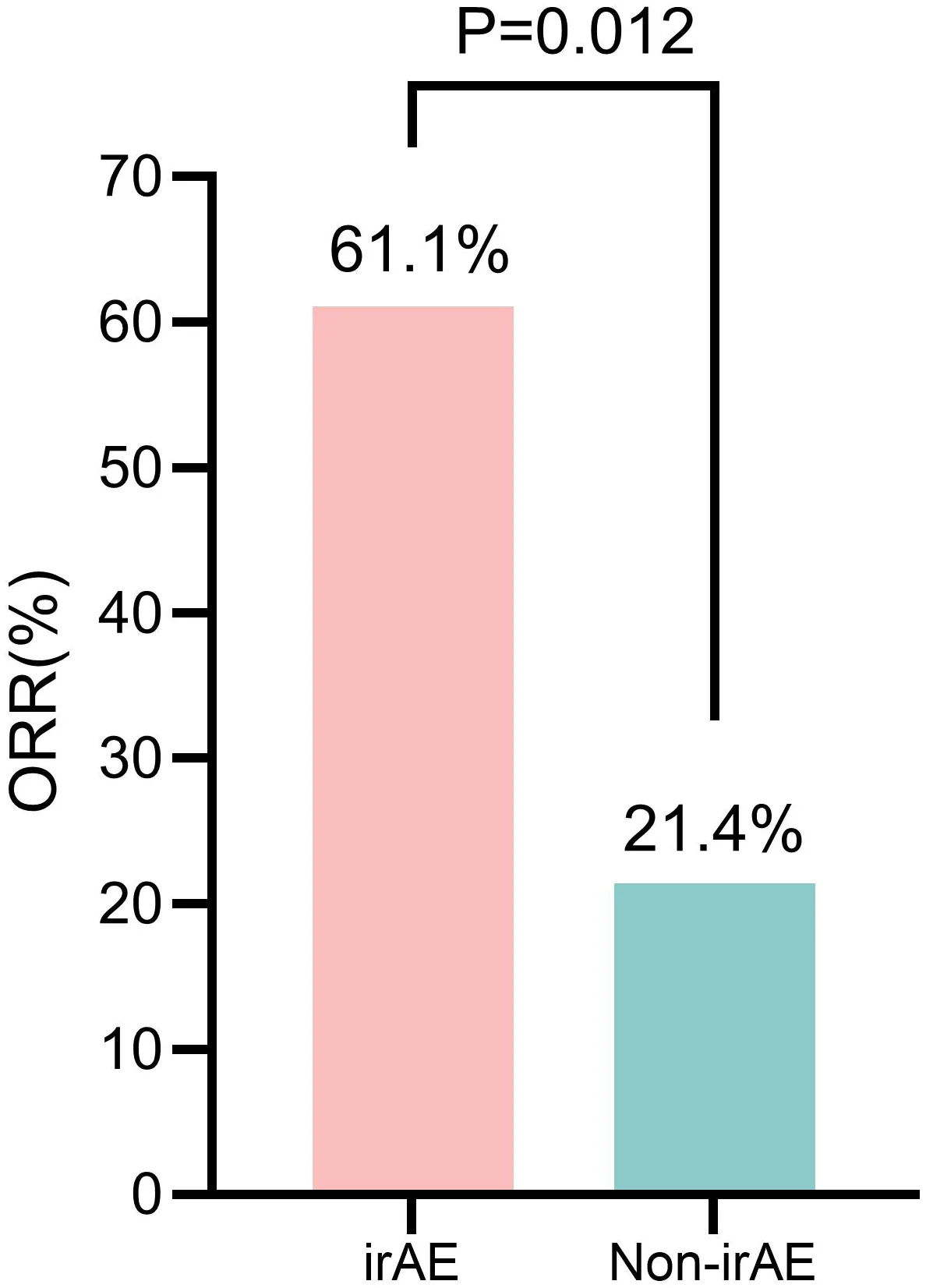
Comparison of objective response rate (ORR) between the irAE group and non-irAE group.

### 3.4 Factors associated with objective response rate

Univariate logistic regression analysis showed that irAE occurrence and treatment cycles were significantly associated with ORR. Patients with irAEs were more likely to achieve an objective response than those without irAEs (OR = 5.762, 95% CI: 1.557–21.328, P = 0.009). Furthermore, an increased number of treatment cycles was associated with a higher likelihood of achieving ORR (OR = 1.359, 95% CI: 1.058–1.745, P = 0.016). ORR was not significantly associated with age, sex, primary tumor type, histological classification, clinical stage, Ki-67 level, treatment line, previous surgery, or combined treatment modalities (**Table 6**). In the exploratory multivariable logistic regression model including irAE occurrence and the number of treatment cycles, the association between irAE occurrence and ORR was attenuated and did not reach statistical significance (OR = 3.847, 95% CI: 0.961–15.410, P = 0.057). After adjustment, the association between the number of treatment cycles and ORR was also not statistically significant (OR = 1.281, 95% CI: 0.980–1.675, P = 0.070) (**Figure 4**).

**Table 6:** Factors associated with objective response rate analyzed by univariate logistic regression analysis.

Variable	OR (95% CI)	P value
Age (≥65 years vs. <65 years)	1.490 (0.433–5.121)	0.527
Sex (Male vs. Female)	0.764 (0.199–2.934)	0.695
Primary tumor type		
Lung cancer vs. Other tumors	2.292 (0.420–12.501)	0.338
Esophageal cancer vs. Other tumors	3.300 (0.692–15.740)	0.134
Histological classification (Neuroendocrine origin vs. Non-neuroendocrine origin)	0.543 (0.162–1.826)	0.324
Clinical stage (IV vs. I–III)	1.689 (0.498–5.732)	0.401
Ki-67 level (continuous variable)	1.026 (0.981–1.072)	0.263
Treatment line (Second-line vs. First-line)	1.957 (0.348–11.008)	0.446
Treatment cycles (continuous variable)	1.359 (1.058–1.745)	0.016
Previous surgery (Yes vs. No)	0.196 (0.022–1.759)	0.146
Combined with chemotherapy (Yes vs. No)	1.185 (0.099–14.136)	0.893
Combined with targeted therapy (Yes vs. No)	1.477 (0.337–6.474)	0.605
Combined with radiotherapy (Yes vs. No)	1.310 (0.341–5.031)	0.695
irAE occurrence (Yes vs. No)	5.762 (1.557–21.328)	0.009

OR, Odds ratio; CI, confidence interval; irAE, immune-related adverse events.

**Figure 4:**
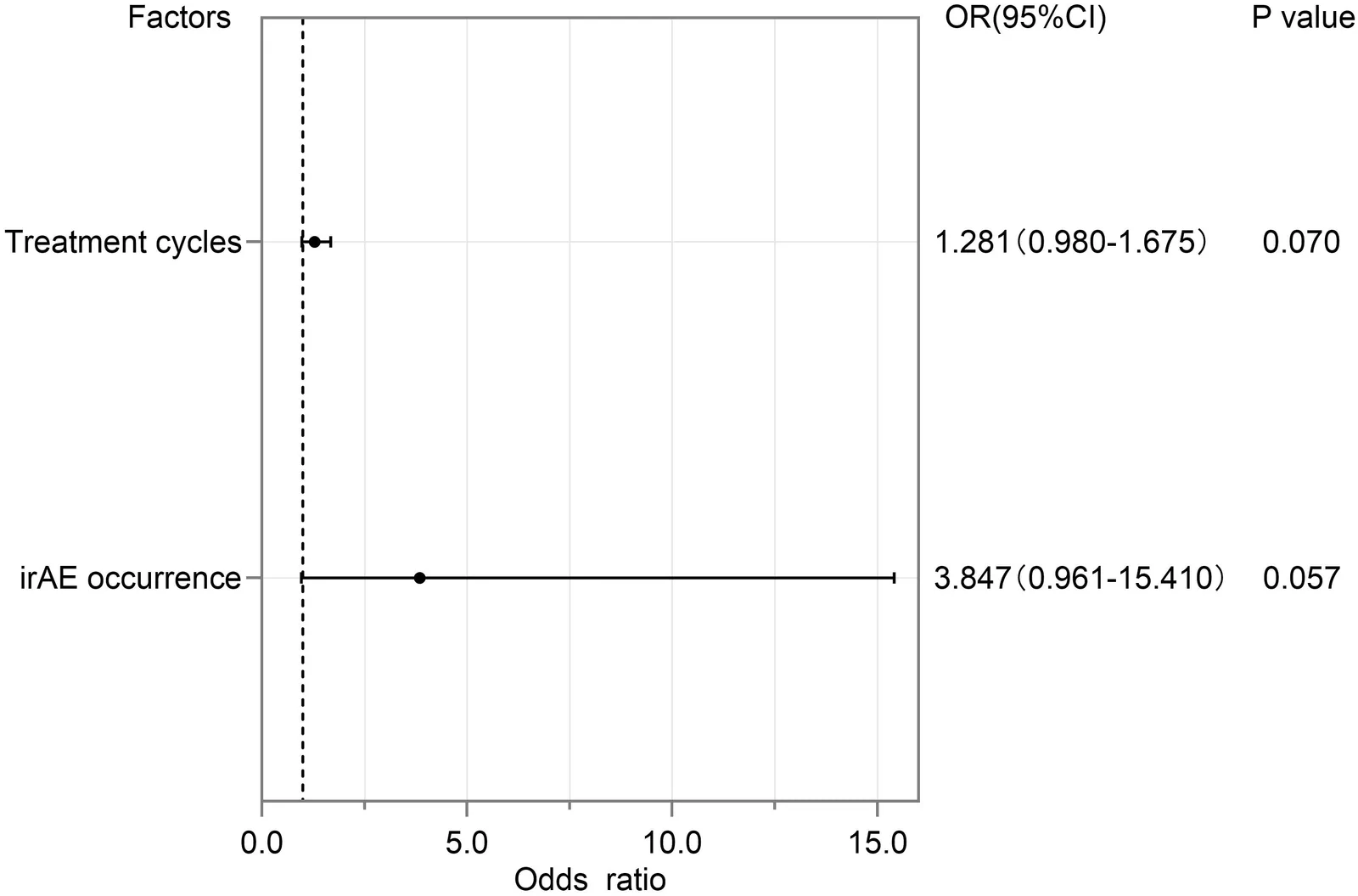
Forest plot of factors associated with ORR analyzed by exploratory multivariable logistic regression analysis.

### 3.5 Association between irAEs and progression-free survival

By the end of follow-up, 16 patients had experienced disease progression, including 8 patients (44.4%) in the irAE group and 8 patients (28.6%) in the non-irAE group. Kaplan–Meier survival analysis showed that the median PFS was 15.0 months (95% CI: 7.563–22.437) in the irAE group, whereas the median PFS was not reached in the non-irAE group. No significant difference in PFS was observed between the two groups (P = 0.188) (**Figure 5**). Univariable Cox proportional hazards regression analysis showed no significant association between irAE occurrence and PFS (HR = 1.906, 95% CI: 0.710–5.113, P = 0.200). Further analysis using a time-dependent Cox proportional hazards model also showed no statistically significant association between irAE occurrence and PFS (HR = 2.444, 95% CI: 0.911–6.557, P = 0.076). The Schoenfeld residual test did not indicate a significant violation of the proportional hazards assumption (P = 0.650). Furthermore, a swimmer plot was used to illustrate the PFS duration, best tumor response, disease progression, and time to first irAE onset for all 46 patients (**Figure 6**).

**Figure 5:**
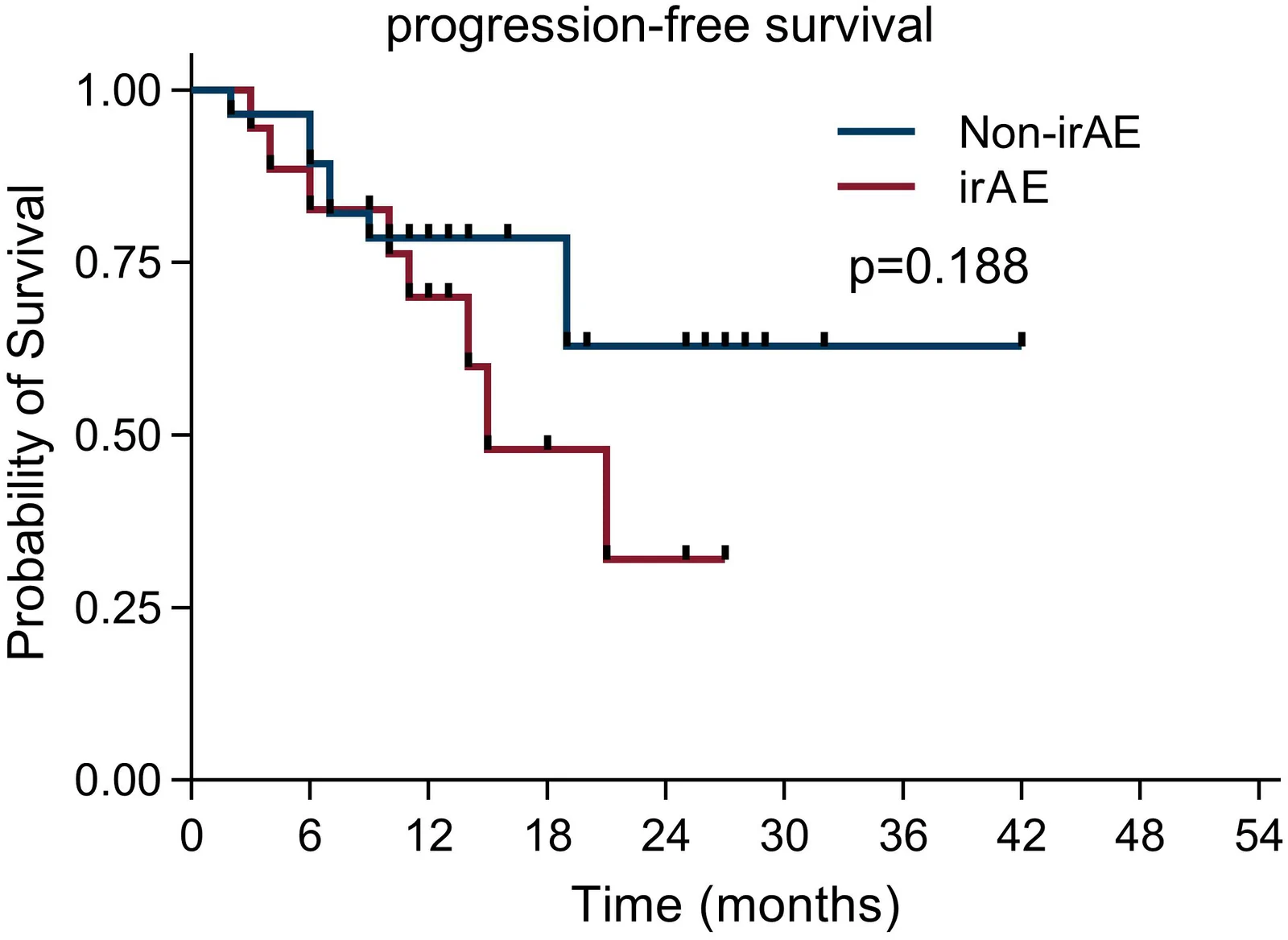
Kaplan–Meier curves for progression-free survival (PFS) according to irAE status.

**Figure 6:**
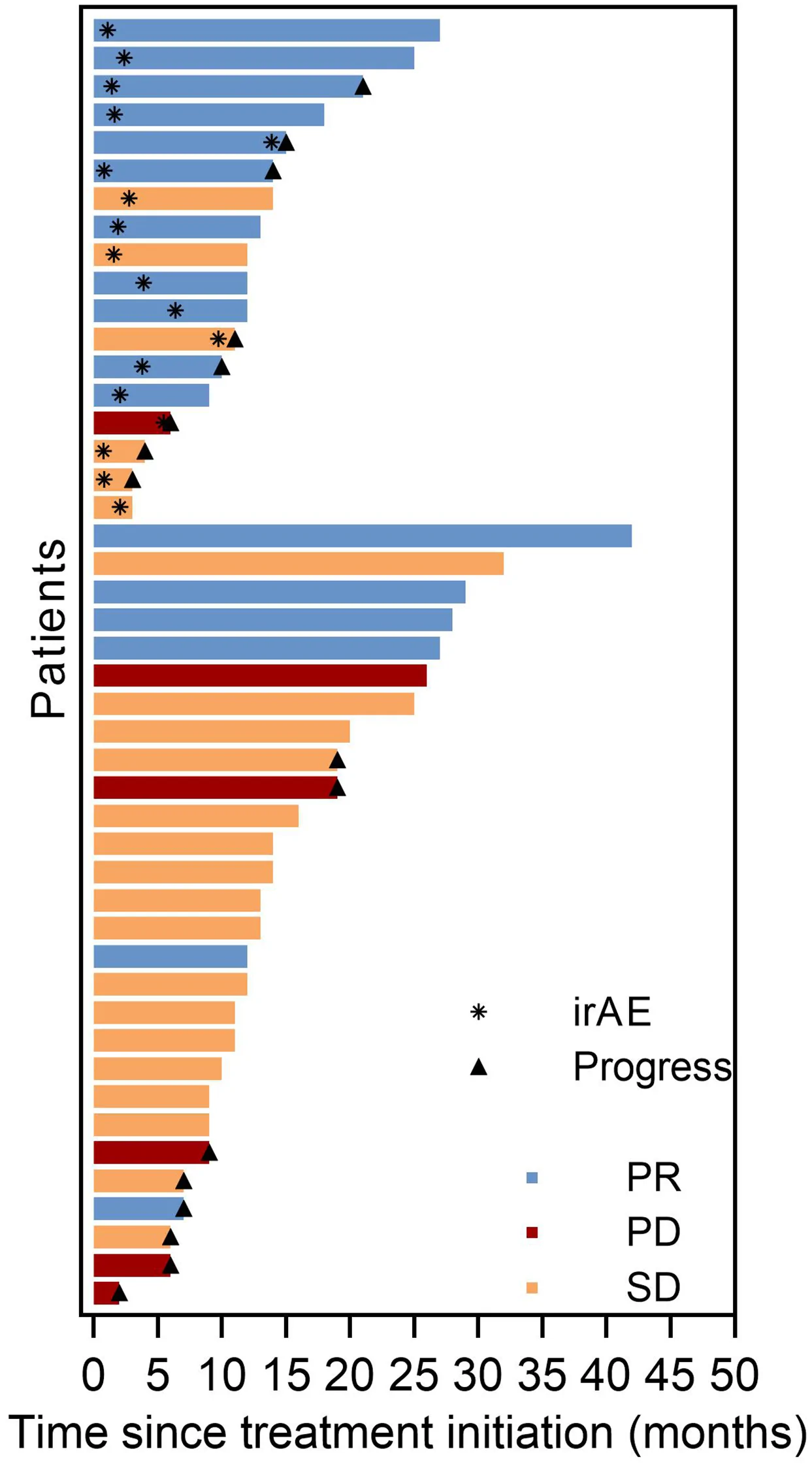
Swimmer plot showing PFS, best overall response, disease progression, and time to first irAE onset in the 46 patients.

## 4 Discussion

Although ICIs have significantly improved clinical outcomes in patients with various solid tumors, treatment-induced immune activation may also lead to the development of irAEs (3, 9). In recent years, irAEs have been recognized not only as manifestations of immune-related toxicity but also as potential markers of effective immune activation (9, 19, 20). Therefore, the potential association between irAEs and the efficacy of ICIs has attracted increasing attention.

This real-world study included 46 patients with solid tumors who received adebrelimab. Among them, 18 patients (39.1%) experienced at least one irAE, most of which were low-grade events. The ORR was higher in the irAE group than in the non-irAE group (61.1% vs. 21.4%); however, after adjustment for the number of treatment cycles, this association was attenuated and did not reach statistical significance (OR = 3.847, 95% CI: 0.961–15.410, P = 0.057). Furthermore, neither conventional Cox regression nor time-dependent Cox analysis accounting for the timing of irAE onset demonstrated a statistically significant association between irAE occurrence and PFS. Therefore, the observed relationship between irAE occurrence and tumor response in this study is more appropriately interpreted as an exploratory observational association, and irAEs cannot be considered independent predictive indicators of adebrelimab efficacy based on these findings alone.

Previous studies have suggested that the development of irAEs may be associated with higher objective response rates and improved survival outcomes following ICI treatment. For example, a meta-analysis of 106 studies involving 41,050 patients with advanced or recurrent lung cancer showed that patients who developed irAEs had a significantly higher ORR than those who did not (relative risk [RR] = 2.03, 95% CI: 1.81–2.28), with RRs of 2.80 and 2.00 for nivolumab- and pembrolizumab-based analyses, respectively (21). A meta-analysis of nivolumab in patients with gastric cancer similarly reported a higher ORR among patients who developed irAEs than among those who did not (24.7% vs. 6.4%; RR = 2.6, 95% CI: 2.0–3.3), along with trends toward improved PFS and OS (22). Furthermore, a pooled analysis of phase III trials of atezolizumab in patients with non-small cell lung cancer reported an ORR of 61% among patients who developed irAEs, compared with 37% among those who did not (23). Similar findings were reported in a recent large-scale meta-analysis of 63,780 patients from 184 studies, in which the ORR was significantly higher in the irAE group than in the non-irAE group (39.4% vs. 16.2%; RR = 2.32, 95% CI: 2.02–2.67), and irAEs were also associated with longer PFS and OS (24). A meta-analysis of patients with hepatocellular carcinoma likewise reported associations between irAEs and ORR, PFS, and OS (25). However, substantial heterogeneity exists across studies with respect to patient populations, tumor types, ICI agents, combination treatment regimens, definitions of irAEs, timing of efficacy assessment, and statistical methods. In addition, time-related factors, such as treatment exposure and the timing of irAE onset, may have influenced the observed associations in some studies. Therefore, the associations between irAEs and treatment benefit reported in previous studies should be interpreted cautiously and should not be considered evidence that irAEs are reliable and independent predictors of treatment efficacy.

The higher ORR observed in the irAE group in the present study was directionally consistent with findings from previous studies; however, after further adjustment for treatment exposure, this association was attenuated and no longer statistically significant. Patients in the irAE group received significantly more treatment cycles than those in the non-irAE group [6 (5–8) vs. 5 (2–6) cycles, P = 0.016]. This finding suggests that treatment exposure may be an important factor underlying the observed association between irAE occurrence and tumor response. Longer treatment duration increases the opportunity for irAEs to develop, but it also provides greater opportunity for patients to receive adequate anticancer therapy and achieve tumor response. Therefore, simply classifying patients according to whether they experienced an irAE at any point during the entire treatment course may, to some extent, attribute the therapeutic opportunities associated with longer treatment exposure to the occurrence of irAEs. After adjustment for the number of treatment cycles, the association between irAE occurrence and ORR changed from statistically significant to non-significant, suggesting that treatment exposure may have contributed to the observed association between irAEs and treatment efficacy.

Fixed grouping based on whether an irAE occurred during treatment may introduce time-related bias, particularly immortal time bias, in addition to the potential impact of treatment exposure (26). This is because patients must survive and remain on treatment until an irAE occurs to be classified into the irAE group.To reduce the potential impact of this bias on the PFS analysis, we further performed a time-dependent Cox model, in which patients were considered unexposed before the first occurrence of an irAE and exposed thereafter. Neither conventional Cox regression (HR = 1.906, 95% CI: 0.710–5.113, P = 0.200) nor time-dependent Cox analysis (HR = 2.444, 95% CI: 0.911–6.557, P = 0.076) demonstrated a statistically significant association between irAE occurrence and PFS. Given that only 16 PFS events occurred in this study, the effect estimates were subject to considerable uncertainty. Therefore, a definitive statistically significant association between irAE occurrence and PFS cannot be established. Taken together, these findings indicate that the relationship between irAE occurrence and PFS should be interpreted cautiously, and irAEs cannot be considered independent predictors of treatment benefit from adebrelimab based on the present findings.

In terms of safety, 40 patients (87.0%) in the present study experienced at least one TRAE, including 5 patients (10.9%) who experienced grade 3–4 TRAEs. The most common adverse events included decreased white blood cell count, decreased neutrophil count, nausea, anemia, decreased platelet count, decreased appetite, and fatigue. CAPSTONE-1 was a pivotal phase III trial evaluating adebrelimab plus carboplatin/etoposide in patients with extensive-stage small-cell lung cancer. In that trial, the most common grade 3–4 treatment-related adverse events in the adebrelimab plus chemotherapy group were decreased neutrophil count (76%), decreased white blood cell count (46%), decreased platelet count (38%), and anemia (28%) (10). Compared with CAPSTONE-1, the incidence of grade 3–4 hematologic adverse events was numerically lower in the present study. However, direct comparisons of the safety profiles of adebrelimab between the two studies are inappropriate due to differences in tumor types, treatment regimens, and study designs. In particular, most patients in the present study received combination therapy, and some hematologic and gastrointestinal toxicities may have been primarily attributable to concomitant chemotherapy. In the present study, 18 patients (39.1%) experienced irAEs, including 3 patients (6.5%) with grade 3–4 irAEs, whereas the incidences of irAEs and grade ≥3 irAEs in CAPSTONE-1 were 28% and 5%, respectively (10). Hypothyroidism was among the common irAEs in both studies. Although the overall incidence of irAEs was numerically higher in the present study, most events were low grade. Given the differences in patient populations and treatment regimens, these findings should be interpreted primarily as a contextual comparison. They should not be taken as evidence of a substantive difference in the safety profile of adebrelimab between the two studies.

The present study included patients with heterogeneous tumor types and treatment regimens, including lung cancer, esophageal cancer, neuroendocrine tumors, and other malignancies, as well as different lines of therapy and combination treatment regimens. This heterogeneity increases the complexity of interpreting the efficacy outcomes. Different tumor types and combination treatment regimens may simultaneously influence both tumor response and the occurrence of irAEs; therefore, the observed differences in ORR may not be entirely attributable to irAE occurrence. Given the limited sample size, we did not perform further stratified analyses by tumor type to avoid unstable estimates resulting from small subgroup sizes. Therefore, the findings of this study should primarily be regarded as exploratory observations in a real-world setting.

The present study has several limitations. First, as this was a single-center retrospective study with a limited sample size, selection bias, information bias, and unmeasured confounding may have been present. Only 17 patients achieved an objective response and 16 experienced PFS events; the limited number of outcome events reduced statistical power and may have resulted in unstable estimates in the regression models. Therefore, all related analyses should be considered exploratory. Second, the study included various types of solid tumors, with some heterogeneity in treatment lines and combination treatment regimens. This heterogeneity may have influenced the efficacy and safety outcomes and limited the generalizability of the findings. Furthermore, comprehensive immune and biomarker data were unavailable, and serial assessments of target lesions were incomplete in some patients. Therefore, we were unable to further investigate the potential biological mechanisms underlying the association between irAEs and antitumor response or the dynamic changes in tumor burden. Although a time-dependent Cox model was used to account for the time-dependent nature of irAE occurrence, the limited number of PFS events still resulted in limited statistical power and stability of the analysis. Future studies should involve larger, multicenter, prospective cohorts with relatively standardized treatment regimens and systematic recording of irAE onset, treatment exposure, and efficacy assessments. Time-dependent or landmark analyses should be employed to appropriately account for the time-dependent nature of irAEs. In addition, systematic assessment of immune and molecular biomarkers, including PD-L1 expression, tumor mutational burden, and the tumor immune microenvironment, should be incorporated to further clarify the association between irAEs and treatment benefit from adebrelimab and to explore the underlying biological mechanisms (27–30).

## 5 Conclusion

In this study, 39.1% of patients with solid tumors treated with adebrelimab experienced irAEs, most of which were low-grade events. Although the irAE group had a higher ORR, this association did not reach statistical significance after adjustment for the number of treatment cycles. In addition, time-dependent Cox analysis did not demonstrate a statistically significant association between irAE occurrence and PFS. Therefore, the present study does not provide sufficient evidence to support irAEs as independent predictors of adebrelimab efficacy. Larger, multicenter, prospective studies are needed to further validate the relationship between irAEs and treatment benefit from adebrelimab.

## Data Availability

The raw data supporting the conclusions of this article will be made available by the authors, without undue reservation.
